# Age Differences in the Effect of Framing on Prosocial Giving Decisions

**DOI:** 10.1093/geroni/igaf122.1402

**Published:** 2025-12-31

**Authors:** Enna Chen, Wei Xing Toh, Yochai Shavit, Laura Carstensen

**Affiliations:** Stanford University, Stanford, California, United States; Nanyang Technological University, Singapore, Singapore; Stanford University, Stanford, California, United States; Stanford University, Stanford, California, United States

## Abstract

According to the Federal Reserve, Americans ages 55 and over hold an increasing percentage of US wealth, reaching 73.2% in 2024 compared with 55.8% in 2000. Promoting charitable giving across the adult life span, therefore, presents significant opportunities to improve communities. Although older adults are more prosocial than younger adults, consistent with Socioemotional Selectivity Theory, they also give more selectively to familiar causes and recipients, challenging efforts to increase charitable giving. Based on findings of age differences in the framing effect on health decision making, the present study examines the effect of positive and negative framing on older and younger adults’ prosocial giving decisions. Participants (N = 293) aged 40 to 86 were randomly assigned to one of three conditions: positive framing (vignette emphasizing beneficial outcomes of donations), negative framing (vignette emphasizing adverse consequences of donor inaction), and a neutral condition (no vignette). Participants then allocated a hypothetical windfall between education funds for younger relatives and under-resourced non-kin. Age was positively associated with giving to non-kin. Although there was no main effect of framing, a significant interaction effect between age and framing indicates that negative framing is more persuasive for older adults than middle-aged adults. Furthermore, older adults rated negative framing more positively and less negatively than middle-aged adults. Findings suggest that older adults may perceive the negative information less as a source of distress and more as an opportunity to assist. Understanding these differences may inform strategies to encourage prosocial behavior across different age groups.

